# Variability of three‐dimensional knee morphology cannot be effectively assessed using a coronal plane knee alignment classification in total knee arthroplasty patients

**DOI:** 10.1002/jeo2.70039

**Published:** 2024-10-26

**Authors:** Ishaan Jagota, Joshua Twiggs, Brad Miles, Jonathan V. Baré

**Affiliations:** ^1^ Research and Development 360 Med Care Sydney New South Wales Australia; ^2^ Research and Development Enovis ANZ Sydney New South Wales Australia; ^3^ College of Science and Engineering Flinders University Adelaide South Australia Australia; ^4^ Melbourne Orthopaedic Group Melbourne Victoria Australia

**Keywords:** CPAK classification system, knee phenotypes, preoperative planning, three‐dimensional knee anatomy, total knee arthroplasty

## Abstract

**Purpose:**

Optimal reproduction of the native three‐dimensional (3D) alignment in total knee arthroplasty (TKA) influences outcomes; however, much of the modern TKA alignment research, such as the coronal plane alignment of the knee (CPAK), focuses only on coronal alignment. Tibial, femoral and tibiofemoral measurements on the axial and sagittal planes were evaluated for their relationship to the arithmetic hip‐knee‐ankle angle (aHKA) and joint‐line obliquity (JLO). These 3D anatomical measurements are also evaluated across CPAK groups.

**Methods:**

A retrospective analysis of the 360 Med Care computed tomography (CT) database was performed. Patient CT scans were segmented and landmarked. Linear regression analysis compared 12 axial and sagittal plane measurements (representing tibial, femoral and tibiofemoral rotation, tibial slope and femoral flexion) with both aHKA and JLO. Nonparametric tests assessed these anatomical measurements across the different CPAK groups, while Cohen's delta (*d*) determined the effect size.

**Results:**

With a sample size of 7450 osteoarthritic knees, significant but weak relationships (*r* < 0.30) were observed between all 12 anatomical measurements and both aHKA and JLO. Tibiofemoral rotations between Insall's axis and both the posterior condylar and the surgical transepicondylar axes demonstrated large effect sizes (*d* > 0.80). However, trivial to small effect sizes (*d* < 0.50) were broadly observed across the 12 axial and sagittal measurements, underscoring their limited clinical significance.

**Conclusions:**

While useful for describing coronal knee anatomy, CPAK phenotypes fail to differentiate tibial, femoral and tibiofemoral rotation, tibial slope or femoral flexion—crucial aspects of 3D surgical planning. Therefore, more comprehensive knee phenotyping solutions are required to guide individualised TKA alignment strategies.

**Level of Evidence:**

Level II.

**Abbreviations**
2D: two‐dimensional
3D: three‐dimensional
aHKA: arithmetic hip‐knee‐ankle
AP: antero‐posterior
CPAK: coronal plane alignment of the knee
CT: computed tomography
HKA: hip‐knee‐ankle
IQR: interquartile range
JLO: joint‐line obliquity
LDFA: lateral distal femoral angle
MPTA: medial proximal tibial angle
PCA: posterior condylar axis
sTEA: surgical transepicondylar axis
TEA: transepicondylar axis
TKA: total knee arthroplasty

## INTRODUCTION

Knee phenotyping classification systems guide alignment strategy by considering patient‐specific anatomy, but they predominantly focus on the coronal plane. Conventional classifications of neutral, varus or valgus oversimplify knee alignment by not considering joint‐line obliquity (JLO) and are influenced by the assessment position (e.g., weight‐bearing or supine) [14, 18]. Moreover, measurements may be influenced by the disease progression of an arthritic knee. In 2019, Hirschmann et al. introduced functional knee phenotypes, which considered the hip‐knee‐ankle (HKA), tibial and femoral coronal angles, identifying 43 phenotypes in a healthy population [11]. While useful for alignment strategy, this system is complex and limited by HKA assessment, which is influenced by assessment position and osteoarthritis‐related deformity [13]. MacDessi et al.'s coronal plane alignment of the knee (CPAK) classification system addresses these limitations by considering constitutional coronal limb alignment [19], which remains constant despite joint space narrowing. CPAK classifies knees into nine phenotype groups using arithmetic HKA (aHKA) and JLO [18]. Although CPAK phenotype distribution varies with gender and patient ethnicity [9, 12, 22, 23, 30], it is used as an operative alignment strategy to guide joint balance optimisation [8, 19]. As a simple and pragmatic tool, CPAK was developed using two‐dimensional (2D) antero‐posterior (AP) radiographs, thus restricting knee phenotyping to the coronal plane.

However, TKA outcomes are influenced by three‐dimensional (3D) anatomical variability. For instance, distal femoral rotation variability [32] complicates the use of a standard 3° rule for external rotation of the transepicondylar axis (TEA) relative to the posterior condylar axis (PCA) [31]. While tibial tubercle referencing rotations display high variability [4], excessive tibiofemoral internal rotation or rotational mismatch can result in inferior postoperative pain and functional outcomes [2, 3, 16, 25]. Interpatient [21] and intrapatient [5, 10, 20] tibial slope variability also complicate TKA planning. Due to such anatomical variabilities, standardised rotational and sagittal alignment strategies may not be suitable for all patients [1]. Therefore, it is important to understand the relationship between CPAK parameters and axial and sagittal plane variables.

Corbett et al. investigated the relationship between rotational and sagittal measurements and aHKA and JLO and evaluated these variables across CPAK groups [7]. However, their analysis was limited to five anatomical variables and a moderate TKA population size. This study expands on their work by examining a broader range of axial and sagittal characteristics in a considerably larger patient cohort.

Our primary aim is to investigate the linear relationship between 12 common axial and sagittal measurements and aHKA and JLO in a large TKA patient cohort. We also evaluate how these anatomical measurements vary across CPAK groups. We hypothesise limited relationships between the axial and sagittal measurements and both aHKA and JLO, with few clinically applicable differences across CPAK groups.

## METHODS

A retrospective analysis of the 360 Med Care computed tomography (CT) database was performed, composed of patients from 107 different orthopaedic surgeons who were undergoing TKA between 17 August 2015 and 8 January 2024. Joints undergoing revision or complex primary surgery, wherein revision prostheses was required, were excluded from the study.

**Figure 1:**
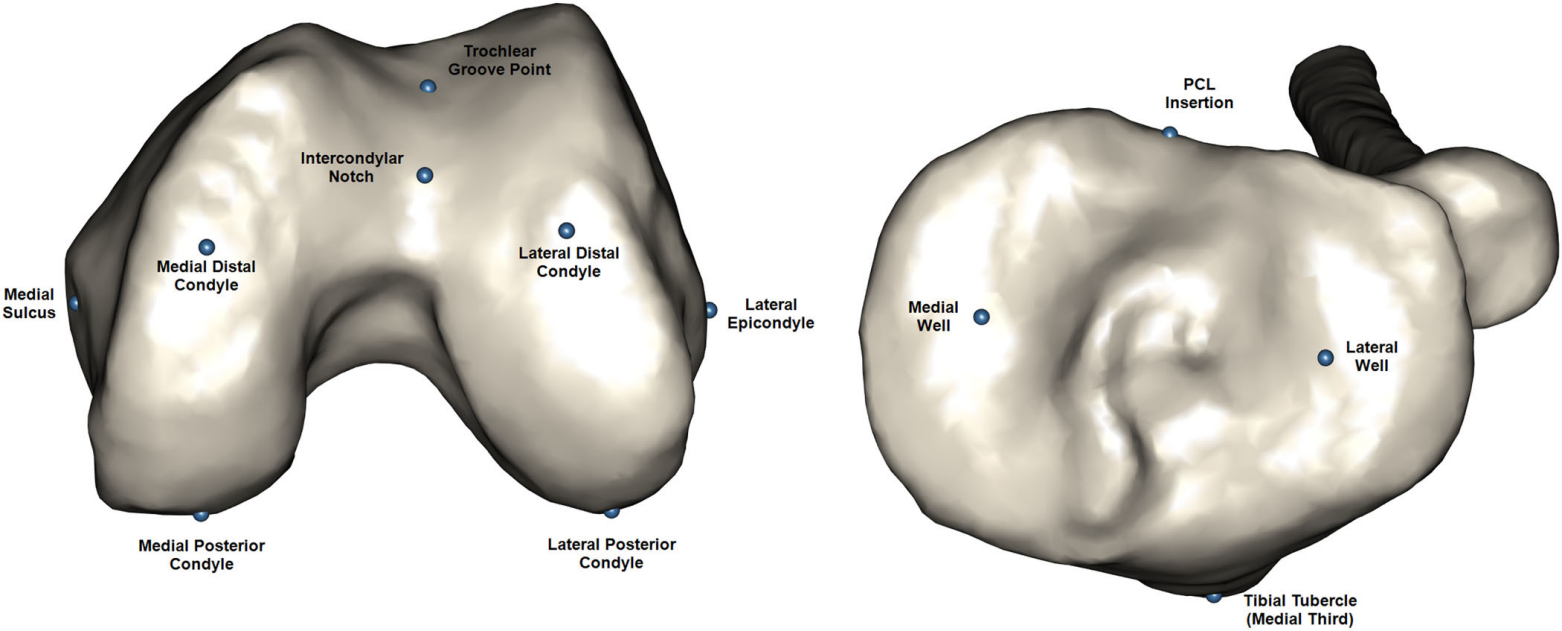
Representation of the key landmarks on the distal femur and proximal tibia. PCL, posterior cruciate ligament.

All patients received a bilateral lower‐limb preoperative CT scan with a maximum slice thickness of 1.25 mm. The CT scans were segmented and landmarked by engineers using Simpleware ScanIP (Synopsys, Inc.) Figure 1.

The final bone models and landmarks were used to calculate a range of measurements with a focus on coronal, axial and sagittal measurements as outlined and defined in Table 1. On the coronal plane, the proximal tibial angle (MPTA) and lateral distal femoral angle (LDFA) were calculated and used to determine the aHKA (MPTA–LDFA) and JLO (MPTA + LDFA). The aHKA and JLO measurements were used to define the knee as varus, neutral, or valgus and apex distal, apex neutral, or apex proximal, respectively. By combining the aHKA and JLO definitions for a knee, it was categorised in accordance with CPAK [19].

**Table 1:** Details of the anatomical measurements used for the analyses, including plane of measurement, reference bone(s) and description of the measurement.

Measurement	Plane	Bone reference	Description
Medial proximal tibial angle (MPTA)	Coronal	Tibia	The medial angle between the mechanical axis of the tibia and the line joining the medial and lateral tibial wells.
Lateral distal femoral angle (LDFA)	Coronal	Femur	The lateral angle between the mechanical axis of the femur and the line joining the medial and lateral distal condyles.
Arithmetic hip‐knee‐ankle angle (aHKA)	Coronal	Tibiofemoral	aHKA = MPTA–LDFA The aHKA can be categorised as varus (aHKA < −2°), neutral (−2° ≤ aHKA ≤ 2°) or valgus (aHKA > 2°).
Joint line obliquity (JLO)	Coronal	Tibiofemoral	JLO = MPTA + LDFA The JLO can be categorised as apex distal (JLO < 177°), apex neutral (177° ≤ JLO ≤ 183°) or apex proximal (JLO > 183°).
Tibial torsion	Axial	Tibial	Angle between the line joining the medial and lateral wells and the line joining the medial and lateral malleoli. Positive angle denotes external rotation.
Insall's axis to Cobb's axis	Axial	Tibial	The degree of external rotation of Insall's axis from Cobb's axis. Insall's axis was defined as the line joining the posterior collateral ligament (PCL) insertion point and the medial third of the tibial tuberosity [33]. Cobb's axis was defined as the line joining the medial and lateral condylar centres [6].
sTEA to PCA	Axial	Femoral	The degree of external rotation of the surgical transepicondylar axis (sTEA) from the posterior condylar axis (PCA). sTEA was defined as the line joining the medial sulcus and lateral epicondyle landmarks. PCA was defined as the line joining the medial and lateral posterior condyle landmarks.
Femoral AP axis to sTEA	Axial	Femoral	The degree of external rotation of the femoral anteroposterior (AP) axis from the sTEA. The AP axis was the same as Whiteside's line, was defined as the line joining the deepest part of the trochlear groove and centre of the intercondylar femoral notch [34].
Femoral AP axis to PCA	Axial	Femoral	The degree of external rotation of the femoral AP axis from the PCA.
Insall's axis to sTEA	Axial	Tibiofemoral	The degree of external rotation of Insall's axis from the sTEA with consideration to tibiofemoral position during the computed tomography (CT) scan.
Insall's axis to PCA	Axial	Tibiofemoral	The degree of external rotation of Insall's axis from the PCA with consideration to tibiofemoral position during the CT scan.
Cobb's axis to sTEA	Axial	Tibiofemoral	The degree of external rotation of Cobb's axis from the sTEA with consideration to tibiofemoral position during the CT scan.
Cobb's axis to PCA	Axial	Tibiofemoral	The degree of external rotation of the Cobb's axis from the PCA with consideration to tibiofemoral position during the CT scan.
Medial tibial slope	Sagittal	Tibial	The degree of posterior tibial slope (flexion) of the medial plateau relative to the tibial mechanical axis. The tibial mechanical axis was defined as the line joining the centre of the medial and lateral malleoli to the centre of the PCL insertion and the medial third of the tibial tubercle.
Lateral tibial slope	Sagittal	Tibial	The degree of posterior tibial slope (flexion) of the lateral plateau relative to the tibial mechanical axis.
Femoral bow flexion	Sagittal	Femoral	The angle between the femoral mechanical axis and the line joining the distal femur centre to the mid‐femur centre. This was used as a proxy for femoral bow flexion. Positive angle denotes flexion. The femoral mechanical axis was defined as the line joining the femoral head centre to the distal femur centre. The mid‐femur centre was the point at the centre of femur at the cross‐section halfway between the distal femur centre and femoral head centre landmarks.

*Note*: Apart from the CPAK constituents, these specific measurements were used as they were routinely available from the 3D analyses performed during TKA planning for each patient within the study cohort.

Consistent with the literature, CPAK types VII, VIII and IX were excluded from all statistical analysis as they comprised a small portion of the population [19]. Descriptive statistics, including mean and standard deviation and interquartile range (IQR), were used to characterise all anatomical measurements described in Table 1 across the full study population, as well as each CPAK type. Pearson's correlation coefficients were used to investigate the linear relationship between the aHKA and JLO with each of the axial and sagittal anatomical measurements, with *p* < 0.05 denoting statistical significance. The normality of the data across the cohort and each CPAK group was tested subjectively using histograms and Q–Q plots and objectively with measures of skewness and kurtosis. Due to nonparametric distributions, Kruskal–Wallis tests were performed to compare the mean values of the axial and sagittal measurements across the different phenotypes. Where statistical significance was observed, post hoc Mann–Whitney *U* was used to identify which pairs of phenotypes displayed significantly different distributions for the measurement. The *p*‐values obtained from the post hoc analysis were adjusted using the Bonferroni method, with an adjusted significance of *p* < 0.0033. For each axial and sagittal measurement, Cohen's delta (*d*) was used to measure the absolute standardised mean difference between each pair of phenotypes, where a larger value would denote a greater effect size. Values of <0.2, 0.2–0.5, 0.5–0.8 and >0.8 were deemed trivial, small, moderate and large, respectively. All statistical analyses were performed in Posit R Studio.

## RESULTS

Demographic characteristics and CPAK distributions for the 7450 knees from 6235 patients are outlined in Table 2. The most common group was type II (apex distal JLO with a neutral aHKA), representing 34.6% of the study cohort. Types I (31.8%) and III (21.0%) were the next most frequent groups. In descending order, types I (38.3%), II (33.1%) and III (13.8%) were most prevalent amongst men and types II (35.0%), III (27.0%) and I (26.5%) were most common among women.

**Table 2:** Patient demographics for the full study cohort and for the men and women subpopulations.

Measurement	All (*n* = 7450)	Men (*n* = 3336)	Women (*n* = 4114)
Left/right	3475/3975	1572/1764	1903/2211
Age (years)	69.7 (IQR 11.6)	68.8 (IQR 11.7)	70.4 (IQR 11.4)
CT HKA (° Varus)	4.0 (IQR 7.2)	5.2 (IQR 5.8)	2.9 (IQR 8.2)
MPTA (°)	86.3 (IQR 3.7)	86.0 (IQR 3.6)	86.5 (IQR 3.6)
LDFA (°)	86.8 (IQR 3.3)	87.3 (IQR 3.2)	86.4 (IQR 3.3)
aHKA (°)	−0.5 (IQR 5.2)	−1.3 (IQR 4.8)	0.2 (IQR 5.2)
JLO (°)	173.1 (IQR 4.6)	173.3 (IQR 4.7)	172.9 (IQR 4.5)
CPAK type			
I	2370 (31.8%)	1279 (38.3%)	1091 (26.5%)
II	2577 (34.6%)	1104 (33.1%)	1473 (35.8%)
III	1567 (21.0%)	459 (13.8%)	1108 (27.0%)
IV	261 (3.5%)	158 (4.7%)	103 (2.5%)
V	324 (4.3%)	171 (5.1%)	153 (3.7%)
VI	282 (3.8%)	115 (3.4%)	167 (4.1%)
VII	20 (0.3%)	13 (0.4%)	7 (0.2%)
VIII	11 (0.1%)	9 (0.3%)	2 (0.0%)
IX	38 (0.5%)	28 (0.8%)	10 (0.2%)

Abbreviations: aHKA, arithmetic hip‐knee‐ankle angle; CPAK, coronal plane alignment of the knee; CT, computed tomography; HKA, hip‐knee‐ankle angle; IQR, inter‐quartile range; JLO, joint line obliquity; LDFA, lateral distal femoral angle; MPTA, medial proximal tibial angle.

Weak linear correlations were observed between the axial and sagittal measurements assessed and the aHKA and JLO (Table 3). Axially, the femoral AP axis to surgical TEA (sTEA) angle displayed the strongest relationship to aHKA (*r* = −0.14, *p* < 0.001) and the Insall's axis to PCA measurement had the strongest correlation to JLO (*r* = −0.28, *p* < 0.001). Of the sagittal measurements, medial tibial slope displayed the strongest relationship to both aHKA (*r* = −0.19, *p* < 0.001) and JLO (*r* = −0.21, *p* < 0.001).

**Table 3:** Correlations of the axial and sagittal anatomical measurements to the arithmetic hip‐knee‐ankle angle (aHKA) and joint‐line obliquity (JLO).

Anatomical measurement	Bone reference	Plane	aHKA			JLO		
			*r*	*p*	Slope	*r*	*p*	Slope
Tibial torsion	Tibial	Axial	0.05	<0.001	0.03	−0.06	<0.001	−0.03
Insall's axis to Cobb's axis	Tibial	Axial	−0.05	<0.001	−0.06	−0.23	<0.001	−0.22
sTEA to PCA	Femoral	Axial	0.03	0.008	0.07	−0.13	<0.001	−0.25
Femoral AP axis to sTEA	Femoral	Axial	−0.14	<0.001	−0.12	0.14	<0.001	0.10
Femoral AP axis to PCA	Femoral	Axial	−0.13	<0.001	−0.11	0.09	<0.001	0.07
Insall's axis to sTEA	Tibiofemoral	Axial	−0.13	<0.001	−0.10	−0.24	<0.001	−0.17
Insall's axis to PCA	Tibiofemoral	Axial	−0.12	<0.001	−0.09	−0.28	<0.001	−0.19
Cobb's axis to sTEA	Tibiofemoral	Axial	0.10	<0.001	0.09	0.09	<0.001	0.07
Cobb's axis to PCA	Tibiofemoral	Axial	0.09	<0.001	0.08	0.14	<0.001	0.11
Medial tibial Slope	Tibial	Sagittal	−0.19	<0.001	−0.15	−0.21	<0.001	−0.15
Lateral tibial Slope	Tibial	Sagittal	0.03	0.004	0.03	−0.04	<0.001	−0.03
Femoral bow flexion	Femoral	Sagittal	0.02	0.077	0.06	0.07	<0.001	0.19

Abbreviations: AP, antero‐posterior; PCA, posterior condylar axis; sTEA, surgical trans‐epicondylar axis.

**Figure 2:**
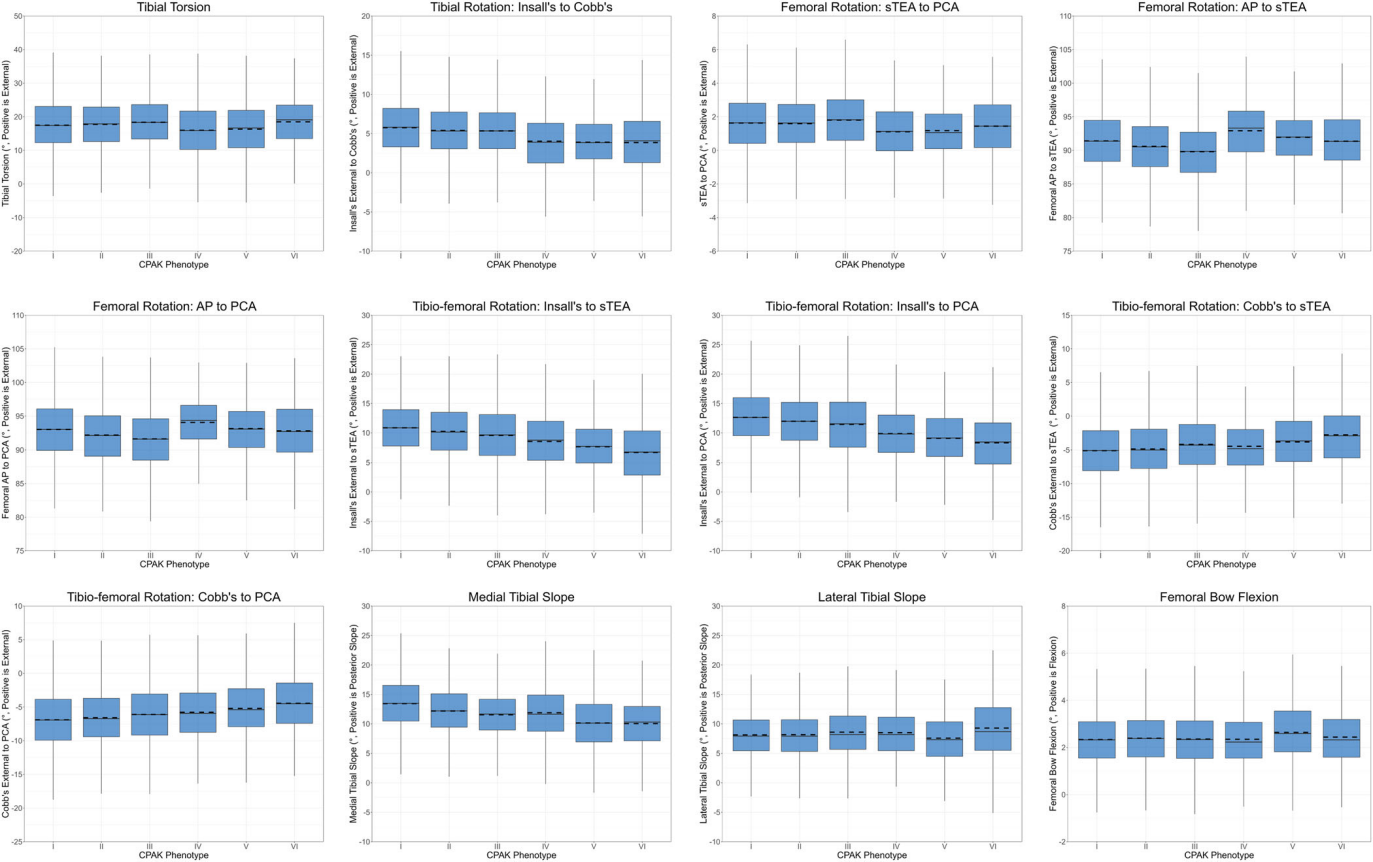
Boxplots displaying the spread of values for the 12 axial and sagittal anatomical measurements across the six coronal plane alignment of the knee phenotypes assessed. Femoral AP, femoral anteroposterior axis; PCA, posterior condylar axis; sTEA, surgical transepicondylar axis.

**Figure 3:**
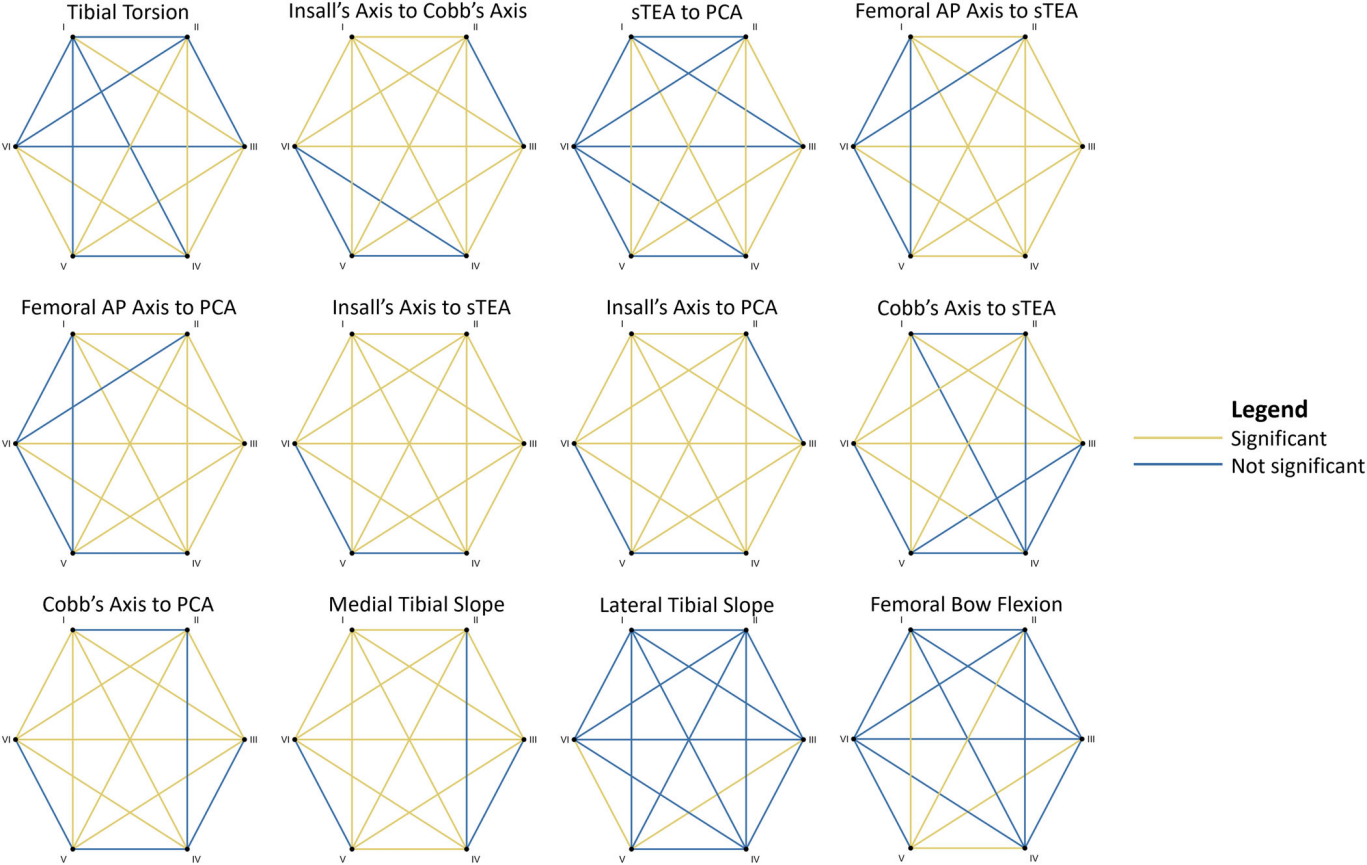
Network plots displaying the post hoc Mann–Whitney *U* test results. After adjusting the *p*‐values using the Bonferroni method, significant results are highlighted in gold and nonsignificant results are in blue. Femoral AP, femoral anteroposterior axis; PCA, posterior condylar axis; sTEA, surgical transepicondylar axis.

The mean values and distributions for the axial and sagittal measurements across the different CPAK phenotypes are displayed in Table 4 and Figure 2, respectively. Table 5 outlines the *d* values for each axial and sagittal measurement across each CPAK group pairing, while Figure 3 summarises the post hoc pairwise comparisons between the CPAK measurements for each phenotype. The largest *d* values were observed between CPAK phenotypes I and VI for the rotation of Insall's axis relative to both the sTEA (0.83) and PCA (0.85). Femoral AP axis to sTEA angle displayed a moderate *d* of 0.68 between CPAK groups III and IV. Sagittally, the medial tibial slope exhibited a moderate *d* (0.66) between CPAK phenotypes I and V, whereas the lateral tibial slope exhibited the lowest maximum *d* (0.23, between groups IV and V) across all anatomical measurements assessed.

**Table 4:** Mean and standard deviation of the axial and sagittal measurements of the full study cohort and each CPAK group from I to VI.

Measurement	Full study cohort (*n* = 7450)	CPAK group					
		I (*n* = 2370)	II (*n* = 2577)	III (*n* = 1567)	IV (*n* = 261)	V (*n* = 324)	VI (*n* = 282)
Tibial torsion (°)	17.71 ± 7.99	17.48 ± 8.24	17.75 ± 7.66	18.39 ± 7.90	15.97 ± 8.53	16.30 ± 7.69	18.50 ± 8.31
Insall's axis to Cobb's axis (°)	5.30 ± 3.66	5.75 ± 3.61	5.40 ± 3.53	5.34 ± 3.58	4.08 ± 3.81	3.90 ± 3.37	3.85 ± 4.25
sTEA to PCA (°)	1.60 ± 1.81	1.63 ± 1.79	1.59 ± 1.76	1.80 ± 1.84	1.13 ± 1.86	1.18 ± 1.76	1.44 ± 1.93
Femoral AP axis to sTEA (°)	90.90 ± 4.79	91.41 ± 4.57	90.62 ± 4.80	89.85 ± 4.87	92.99 ± 4.36	92.03 ± 4.67	91.36 ± 4.49
Femoral AP axis to PCA (°)	92.51 ± 4.83	93.03 ± 4.61	92.22 ± 4.85	91.65 ± 4.96	94.12 ± 4.30	93.20 ± 4.63	92.80 ± 4.76
Insall's axis to sTEA (°)	9.94 ± 5.07	10.86 ± 4.70	10.25 ± 4.95	9.56 ± 5.26	8.53 ± 4.86	7.63 ± 4.87	6.72 ± 5.21
Insall's axis to PCA (°)	11.69 ± 5.25	12.65 ± 4.84	11.98 ± 5.11	11.45 ± 5.46	9.87 ± 4.91	9.00 ± 4.98	8.30 ± 5.36
Cobb's axis to sTEA (°)	−4.64 ± 4.50	−5.11 ± 4.34	−4.85 ± 4.40	−4.22 ± 4.55	−4.45 ± 4.58	−3.74 ± 4.73	−2.87 ± 4.85
Cobb's axis to PCA (°)	−6.39 ± 4.55	−6.90 ± 4.39	−6.59 ± 4.42	−6.11 ± 4.66	−5.79 ± 4.44	−5.11 ± 4.78	−4.45 ± 4.68
Medial tibial slope (°)	12.24 ± 5.01	13.51 ± 5.01	12.17 ± 4.50	11.47 ± 4.75	11.98 ± 5.15	9.97 ± 5.71	10.01 ± 6.05
Lateral tibial slope (°)	8.24 ± 4.93	8.13 ± 4.29	8.13 ± 4.41	8.65 ± 5.24	8.60 ± 4.70	7.22 ± 6.98	8.77 ± 7.93
Femoral bow Flexion (°)	2.37 ± 1.31	2.31 ± 1.33	2.38 ± 1.29	2.35 ± 1.28	2.35 ± 1.48	2.64 ± 1.36	2.42 ± 1.25

Abbreviations: AP, antero‐posterior; CPAK, coronal plane alignment of the knee; PCA, posterior condylar axis; sTEA, surgical trans‐epicondylar axis.

**Table 5:** Cohen's delta (*d*) values for each axial and sagittal measurement between coronal plane alignment of the knee (CPAK) groups.

	II	III	IV	V	VI
A. Tibial torsion					
I	0.03	0.11	0.18	0.15	0.12
II		0.08	0.22	0.19	0.09
III			0.29	0.27	0.01
IV				0.04	0.30
V					0.27
B. Insall's axis to Cobb's axis					
I	0.10	0.11	0.45	0.53	0.48
II		0.02	0.36	0.44	0.40
III			0.34	0.42	0.38
IV				0.05	0.06
V					0.01
C. sTEA to PCA					
I	0.02	0.10	0.27	0.25	0.10
II		0.12	0.25	0.24	0.08
III			0.36	0.35	0.19
IV				0.02	0.16
V					0.14
D. Femoral AP axis to sTEA					
I	0.17	0.33	0.35	0.13	0.01
II		0.16	0.52	0.30	0.16
III			0.68	0.46	0.32
IV				0.21	0.37
V					0.15
E. Femoral AP axis to PCA					
I	0.17	0.29	0.24	0.04	0.05
II		0.11	0.42	0.21	0.12
III			0.53	0.32	0.24
IV				0.21	0.29
V					0.09
F. Insall's axis to sTEA					
I	0.13	0.26	0.49	0.67	0.83
II		0.13	0.35	0.53	0.69
III			0.20	0.38	0.54
IV				0.18	0.36
V					0.18
G. Insall's axis to PCA					
I	0.13	0.23	0.57	0.74	0.85
II		0.10	0.42	0.59	0.70
III			0.31	0.47	0.58
IV				0.18	0.31
V					0.14
H. Cobb's axis to sTEA					
I	0.06	0.20	0.15	0.30	0.49
II		0.14	0.09	0.24	0.43
III			0.05	0.10	0.29
IV				0.15	0.34
V					0.18
I. Cobb's axis to PCA					
I	0.07	0.17	0.25	0.39	0.54
II		0.10	0.18	0.32	0.47
III			0.07	0.21	0.36
IV				0.15	0.29
V					0.14
J. Medial tibial slope					
I	0.28	0.42	0.3	0.66	0.63
II		0.15	0.04	0.43	0.40
III			0.1	0.29	0.27
IV				0.37	0.35
V					0.01
K. Lateral tibial slope					
I	0.00	0.11	0.1	0.16	0.10
II		0.11	0.1	0.16	0.10
III			0.01	0.23	0.02
IV				0.23	0.03
V					0.21
L. Femoral bow flexion					
I	0.05	0.03	0.03	0.24	0.08
II		0.02	0.02	0.20	0.03
III			0.00	0.22	0.06
IV				0.2	0.05
V					0.17

*Note*: The rows and columns correspond to different CPAK groups (I–VI), with *d* values reported at the intersections to indicate the effect size for differences between the respective CPAK group pairings. Effect sizes were categorised as trivial (0.8). For most pairwise comparisons, *d* values indicated a trivial or small effect size, suggesting minimal differences between CPAK groups that are unlikely to be clinically significant.

Abbreviations: AP, anteroposterior; PCA, posterior condylar axis; sTEA, surgical trans‐epicondylar axis.

## DISCUSSION

In this study, most anatomical rotational and sagittal measurements displayed weak linear relationships to both aHKA and JLO. These significantly different findings were attributed to the large sample size, which enhances the statistical power [15]. Furthermore, our analysis highlighted significant differences in axial and sagittal measurements between different CPAK groups. However, the impact of these differences was generally small, as indicated by the *d* values. Notably, large effect sizes were observed only in the tibiofemoral rotations, specifically for the angles between Insall's axis and the sTEA and between Insall's axis and the PCA. This suggests that while CPAK grouping may be associated with variabilities in axial and sagittal planes, it does not effectively distinguish between axial and sagittal characteristics of the knee and is therefore insufficient to describe 3D alignment of the knee during analysis and TKA planning.

Axially, internal rotation of the femoral AP axis relative to sTEA was observed with increased constitutional valgus alignment. This reflects the findings of Luyckx et al. who assessed femoral AP to sTEA rotation relative to HKA [17]. However, clinical implications in TKA require careful consideration. When comparing CPAK group 1 with 3 and 4 with 6, we observe greater femoral external rotation (sTEA to PCA angle) with increasing aHKA. Such an increase in coronal alignment due to either greater tibial or femoral valgus increases the lateral extension joint gap. Meanwhile, greater femoral external rotation decreases the lateral flexion gap and lateralises the femoral trochlea. As a result, the weak statistical finding of internal rotation of femoral AP to sTEA should be treated with caution during TKA as any planned femoral internal rotation can cause extension/flexion gap mismatch. It may therefore be safer to assume no clinical relationship exists, as supported by the lack of a strong effect size (maximum *d* = 0.68) and the findings of Corbett et al., who observed no significant linear relationships between rotational alignment and aHKA or JLO [7].

The external rotation of Insall's axis to both sTEA and PCA displayed weak correlations to aHKA (*r* = −0.14, *p* < 0.001 and *r* = −0.13, *p* < 0.001, respectively) and JLO (*r* = −0.24, *p* < 0.001 and *r* = −0.28, *p* < 0.001, respectively). The latter may be driven by internal tibial rotation (Insall's to Cobb's angle) with an increase in JLO. However, during their assessment of Akagi's line [29] relative to the sTEA in 100 patients, Aglietti et al. observed no significant relationship to coronal long leg deformity [28]. Corbett et al. also employed Akagi's line, measuring its rotation to the PCA [25]. While finding no relationship to aHKA, they observed a significant correlation between tibiofemoral rotation and JLO. This suggests that aligning the tibial component with a fixed reference (e.g., neutral) to Insall's axis may increase the risk of tibial internal rotation or tibiofemoral rotational mismatch as the JLO increases. Nonetheless, the large effect sizes observed for tibiofemoral rotation between CPAK groups I and VI were not mirrored across the other groups. This highlights that the classification system is unable to distinguish tibiofemoral rotational alignment, which is required to support 3D TKA planning.

In the sagittal plane, a weak correlation was observed between medial tibial slope and both aHKA (*r* = −0.19, *p* < 0.001) and JLO (*r* = −0.21, *p* < 0.001), suggesting a decrease in medial posterior tibial slope as either the aHKA becomes more valgus or JLO apex becomes more proximal. Similarly, Panguad et al. observed a significant decrease in medial posterior slope as the coronal alignment categorisation shifted from varus to neutral and neutral to valgus [24]. Their multivariate analysis highlighted that the medial tibial slope was correlated to the HKA (*R* ^2^ = −0.368, *p* < 0.001), while no such relationship was apparent for the lateral tibial slope. Perhaps this is due to the intrapatient variability in medial and lateral tibial slope, reported to be 2.6° on average [20] but greater than 3° for over 45% of osteoarthritic patients [10]. Although intrapatient difference in medial and lateral tibial slope was not assessed in the study, the mean medial and lateral posterior slope values of 12.24° and 8.24°, respectively, provide some insight into the variability between the two compartments. This is emphasised by the lower effect size observed for lateral tibial slope than medial across most CPAK group pairings. The trivial or weak effect sizes observed for both slope measurements suggest that the CPAK classification system cannot guide tibial slope alignment, which is a key component of TKA planning.

The CPAK distribution of this study population varied from the CPAK distribution of an arthritic population as originally reported by MacDessi et al [19]. Most notably, a greater proportion of patients in our study were classified as apex distal than neutral, with a CPAK category of I, II or III. This trend is supported by similar distributions observed in published 3D analyses [7, 29]. Furthermore, Sasaki et al. observed that 2D JLO assessments may be influenced by tibial rotation and knee flexion and demonstrated that 3D joint surface orientation does not correlate to such 2D measurements [27]. Therefore, the difference in CPAK distribution is likely attributable to different imaging modality. We believe that preoperative TKA planning should be performed using 3D joint assessment rather than plain 2D radiographic assessment.

This study had some limitations. The 12 axial and sagittal plane measurements used are nonexhaustive. However, given the lack of consensus on the most suitable references for these tibial, femoral, and tibiofemoral measurements, they do provide an acceptable basis to be used for 3D analysis. They are reflective of commonly used anatomical references; however, we do acknowledge that alternate measurements may yield stronger relationships to aHKA and JLO and/or better differentiate between CPAK phenotypes. Although CPAK types VII, VIII and IX were excluded from statistical analysis, these accounted for just 0.9% of the study cohort. This low proportion reflects distributions observed in regions across the globe [7, 19, 22, 23, 26, 28, 29, 30]. Additionally, the patient population was predominantly Caucasian, so caution must be exercised in generalising these results to other ethnicities.

## CONCLUSION

While the CPAK classification system holds value for describing the knee anatomy in the coronal plane, it displays limited scope in addressing the knee's 3D complexity, thus suggesting the need for more comprehensive classification systems. Future classification systems should be capable of reflecting diverse anatomical variation in all anatomical planes, thereby aiding surgeons’ selection of an appropriate alignment strategy for an individualised TKA. Such classification systems would also add value as a research tool to assess outcomes of TKA relative to 3D alignment. Future research should be directed towards developing and validating such 3D classification systems.

## AUTHOR CONTRIBUTIONS

**Ishaan Jagota**: Conceptualisation; formal analysis; investigation; methodology; project administration; visualisation; writing—original draft. **Joshua Twiggs**: Conceptualisation; methodology; supervision; writing—review and editing. **Brad Miles**: Conceptualisation; resources; supervision; writing—review and editing. **Jonathan V. Baré**: Conceptualisation; methodology; data curation; supervision; writing—review and editing.

## CONFLICTS OF INTEREST STATEMENT

At the time of writing this manuscript, Ishaan Jagota, Joshua Twiggs and Brad Miles were all employees of 360 Med Care, Mathys Orthopaedics and Enovis ANZ. This research was supported by 360 Med Care, Mathys Orthopaedics and Enovis Australia. Brad Miles has stock or stock options in Enovis and 360 Med Care, has/does receive royalties from Enovis ANZ and Corin, and has been a paid consultant for 360 Med Care, Corin and Johnson & Johnson. Jonathan V. Baré receives royalties from Corin, research support from MatOrtho and is a paid speaker for Corin, Enovis, Smith and Nephew, Medacta and MatOrtho. Jonathan V. Baré is the president of Arthroplasty Society of Australia and on committees of the Australian Orthopaedic Association.

## ETHICS STATEMENT

Bellberry Human Research Ethics Committee (study number 201203710).

## Data Availability

Data are not publicly available.
